# Normative data and repeatability for macular ganglion cell layer thickness in healthy Swedish children using swept source optical coherence tomography

**DOI:** 10.1186/s12886-022-02321-1

**Published:** 2022-03-08

**Authors:** Philip Wolf, Eva Larsson, Hanna Åkerblom

**Affiliations:** 1 Department of Ophthalmology, Region Västmanland, Västerås, Sweden; 2 Department of Surgical Sciences, Uppsala University, Uppsala, Sweden; 3 Center for Clinical research, Region Västmanland, Västerås, Sweden

**Keywords:** Macula, Ganglion cell layer, Children, OCT

## Abstract

**Background:**

Optical coherent tomography (OCT) technology is evolving with improved resolution and accuracy in segmentation between different cell layers in the retina. The ganglion cell layer in the macula region is a window to see what is happening in the visual pathways and a macula OCT is an examination that most children tolerate. This makes updated normative data necessary since variables may differ between different OCT devices and normative data for children is often not presented.

The aim of this study was to develop normative data for macular ganglion cell layer thickness in children, measured with swept source OCT, and investigate the repeatability between measurements.

**Methods:**

Healthy Swedish children between 4 and 16 years old, with normal refraction, spherical equivalent mean:1.13 (sd:0.66) dioptre and normal visual acuity Logmar, mean: 0,015 (sd:0,05), were examined with swept source OCT. Macula OCT was performed three times in both eyes and the different retinal layers were evaluated.

**Results:**

Fifty-eight children were screened for inclusion. Fifty-five children were included in the study, 24 boys and 31 girls. Mean age was 8.9 years. Results from right eyes was analysed. The mean average thickness of macular ganglion cell layer thickness, retinal nerve fibre layer/ganglion cell layer boundary to inner plexiform layer/inner nuclear layer boundary, was 68.0 μm (sd:4.0, range:58-77). There was no correlation with sex or age. Fifty-three children manage to complete two, and 41 children three acceptable measurement and the mean coefficient of variation was low.

**Conclusion:**

The ganglion cell layer thickness differs according to which OCT device that is used, and the population examined. This makes normative data essential to accurately interpret results. Swept source OCT of the macula have excellent repeatability and the examination well tolerated in most children making it an investigation useful for diagnosing and following diseases in the optic pathways.

## Background

Optical coherence tomography (OCT) was described in 1991 and introduced in clinical practice 1995 as a cross sectional examination of the retina [1, 2]. Today, OCT is available as a standard examination in most eye clinics. Since 1995 the technology has evolved with improved resolution of examinations which in turn leads to improved accuracy in segmentation between different cell layers in the retina. New OCT devices are continuously being developed and updated normative data is necessary since variables may differ between different OCT devices [3].

In OCT software, available normative data are based on adults. In a paediatric population normative data is of importance to correctly distinguish retinal pathology from normal variations. Previously, several studies have been published using different OCT-devices in children [4–6]. Recently Asian paediatric populations have been studied describing the normative data for children using Triton swept source OCT (Topcon Medical Systems, Inc., Oakland, USA) [7, 8].

In children with diseases affecting the optic nerve and visual pathway ophthalmological examination can be challenging due to compliance issues and especially when it comes to visual field testing using computerized or manual perimetry. In adults, a correlation regarding macular ganglion cell layer (GCL) thickness and visual field abnormalities in glaucoma has been shown [9]. The GCL complex (GCC) is gaining increased interest and has shown signs of retrograde thinning not only after lesions to the optic nerve but also due to occipital lesions [10–12]. Several studies have been made regarding the peripapillary retinal nerve fibre layer (RNFL) in children [13]. However, examining the RNFL and GCL using the macula programme might be easier in children than examining the optic nerve as the latter requires fixating to the side instead of straight forward when performing the OCT-scan. Good correlation between right and left eyes in our setting has earlier been established [14].

The primary aim of this study is to present normative data in a Swedish paediatric population regarding retinal thickness and thickness of GCL using the macular programme with swept source OCT. A secondary aim was to investigate the repeatability of the different measurements.

## Methods

Children aged 4-16 years were invited to participate and examined after obtaining informed consent from the child and their custodians. Recruitment was initialized in participating eye clinics where children were examined by other reasons. They were also recruited by personal communication.

The inclusion criteria were absence of eye disease, uncorrected or best corrected visual acuity of 0.1 LogMAR or better, refraction in cycloplegia not exceeding +/− 3.0 D in spherical equivalent and astigmatism 2.0 D or less. Intraocular pressure was not measured in this study.

Assessments were performed at the Department of Ophthalmology, Västmanland Hospital, Västerås and at the Department of Ophthalmology, Uppsala University Hospital, Uppsala, Sweden. Monocular visual acuity was assessed using digital linear LogMAR charts and if the participant was unable to read, a HVOT chart [15] at 3 m distance was used. Cycloplegia was achieved using eye drops containing cyclopentolate 0,85% and phenylephrine 1,5%. Autorefractor determined cycloplegic refraction. Topcon DRI OCT-1 Triton (Topcon Medical Systems, Inc., Oakland, USA) swept source OCT was used and three consecutive 7 × 7 mm cube 3D Macula OCT-examinations centered on the fovea were performed in each eye, right eye first. If scan was deemed unreliable or showing artefacts it was repeated until three acceptable scans were completed, or the examination was aborted if no further successful scans were to be expected. Data were reviewed using IMAGEnet® 6 (Topcon Medical Systems, Inc., Oakland, USA). Retinal tissue layers were automatically segmented by the Topcon Advanced Boundary Software “TABS™”.

Fifty-eight children were examined for inclusion in the study and 55 children were included. All 55 included participants had at least one OCT-scan of good quality. Quality was assessed by manual review for artefacts (blinking and eye movements) and segmentation errors. The left eye was then examined. No adjustments regarding segmentation were needed in the accepted scans. Fifty-three participants had two scans and 41participants managed the full three scans with good quality of the right eye. Corresponding number of successful exams for the left eye was 52; 45 and 32. We chose to use the results from the right eye for further analysis and in the case of multiple exams averages were used in the calculation of mean values.

Three children did not meet inclusion criteria due to (hyperopia above 3D (*n* = 1), unacceptable examination quality/extensive artefacts in all scans (*n* = 2).

Analysed areas were total average, superior and inferior fraction and in 6 sector grids.

GCIPL is defined as retinal nerve fibre layer/ganglion cell layer boundary (RNFL/GCL) to inner plexiform layer/inner nuclear layer boundary (IPL/INL) and GCC is defined as inner limiting membrane (ILM) to IPL/INL.

### Statistical methods

Data was analysed using IBM SPSS Statistics for Windows, (Version 24.0. Armonk, NY: IBM Corp). Normal distribution was examined using Kolmogorov-Smirnov test. Correlations were assessed Pearson correlation test. Intra-session coefficient of variation (CV), that is the standard deviation divided by the mean, were calculated for all variables. Intraclass correlation (ICC) were calculated for all repeated examinations. A CV close to 0 and ICC 1.0 is regarded as perfect.

## Results

Fifty-five children, (24 boys), met the inclusion criteria with acceptable quality of examinations, see Table 1.

**Table 1:** Descriptive data of the 55 children included in the study (right eyes)

		Mean (SD)	Range
Age	Years	8.9 (3.1)	5 – 16
Visual acuity	logMAR	0.015 (0.05)	−0.1 - 0.1
Refraction SE	Dioptres	1.13 (0.66)	−0.25 - + 2.75

*SE* Spherical equivalent, *SD* standard deviation

**Fig. 1:**
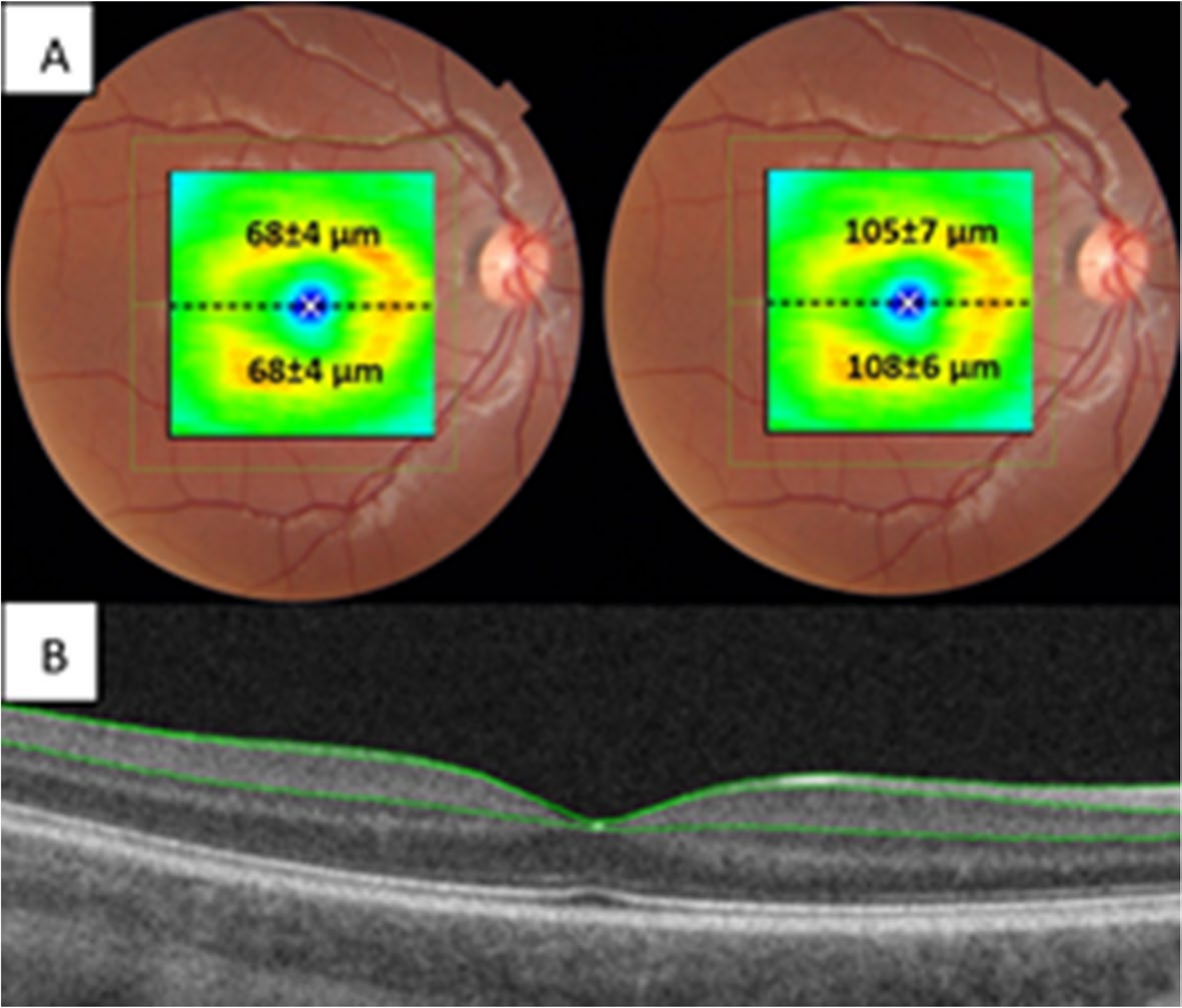
**A**. Macular ganglion cell layer (GCL). Average values of superior/inferior GCL-IPL (top left) and GCC (top right) thickness, 7x7mm square measurement shown in figures **B**. Bottom figure shows segmentation boundaries, ILM (internal limiting membrane) top green line, RNFL/GCL (retinal nerve fibre layer/ganglion cell layer) middle green line, IPL/INL (inner plexiform layer/inner nuclear layer) bottom green line. GCIPL: RNFL/GCL to IPL/INL. GCC: ILM to IPL/INL

Total mean average for GCIPL was 68.0 μm (sd:4.0, range:58-77) and for GCC was 107.1 μm (sd:6.5, range:92-120). Values for superior and inferior GCL thickness, GCIPL, GCC and segmentation lines are presented in Fig. 1.

Retinal thickness, GCIPL and GCC are presented in Table 2.

**Table 2:** Retinal and ganglion cell layer thickness (μm) in 6 sector grid

Sector	Retina ILM-RPE		GCIPL GCL-IPL		GCC ILM-INL	
	Mean(SD)	Range	Mean(SD)	Range	Mean(SD)	Range
1	275(13)	244-309	74(5)	62-85	96(7)	81-121
2	289(12)	261-316	72(5)	60-84	109(7)	81-124
3	299(12)	269-336	76(5)	61-89	119(8)	100-133
4	296(11)	268-329	76(5)	66-87	120(7)	104-135
5	281(12)	250-308	71(4)	62-82	109(7)	95-123
6	277(12)	247-310	76(5)	64-88	101(7)	87-124

*GCC* Ganglion cell complex, *GCL* Ganglion cell layer, *ILM* Internal limiting membrane, *RPE* Retinal pigment epithelium, *IPL* Inner plexiform layer, *INL* Inner nuclear layer

**Fig. 2:**
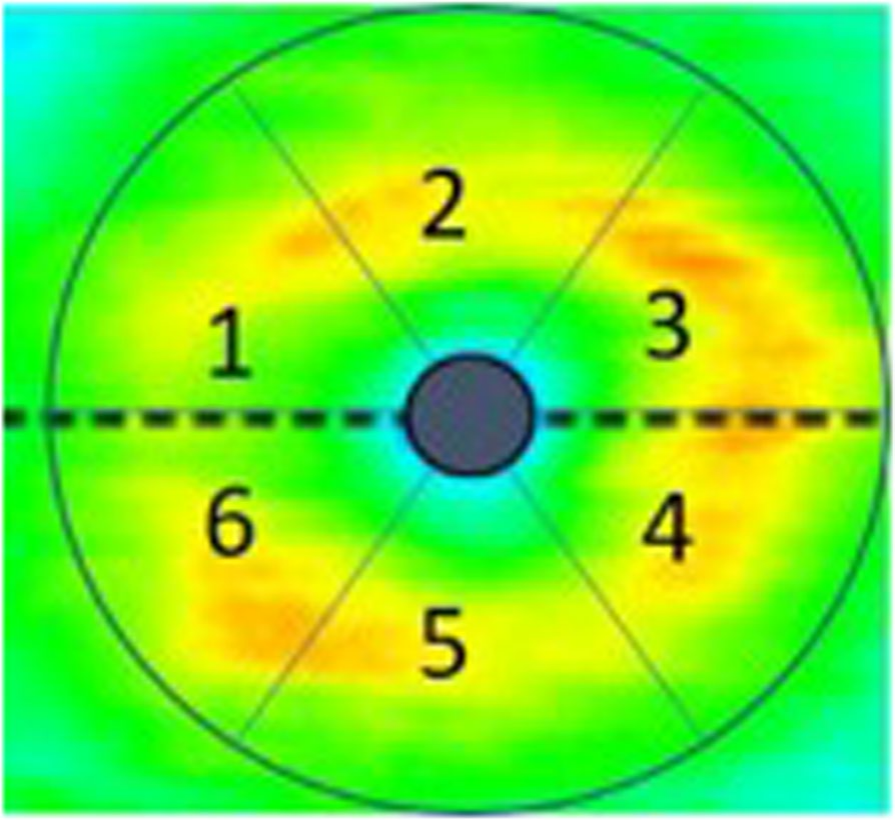
6 sector grid of the macula, right eye

GCIPL and GCC analysed in 6 sector grids are shown in Fig. 2.

There were no correlations with age in GCIPL and GCC, r:0.12, p:0.37 and r:0.22, p:0.11 respectively. There were no correlation with sex and GCIPL and GCC, r:0.02, p:0.89 and r:-0.04, p:0.79 respectively.

**Fig. 3:**
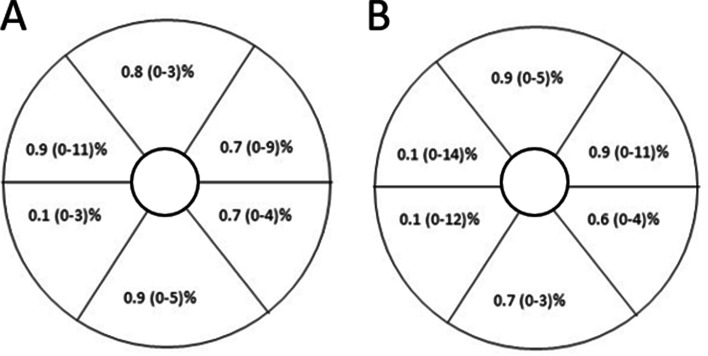
Median and range coefficient of variation in repeated scans for **A**. GCIPL (retinal nerve fibre layer/ganglion cell layer boundary to inner plexiform layer/inner nuclear layer boundary) and **B**. GCC (inner limiting membrane to inner plexiform layer/inner nuclear layer boundary)

Median CV for total average, superior, inferior and the 6 sector grids for GCIPL and GCC was between 0.6 and 1.1%, see Fig. 3. ICC was > 0.9 for all presented variables.

## Discussion

In this study we present normative data for thickness of macular GCL in healthy Swedish children with normal visual acuity and refraction, measured with swept source OCT.

Previous studies have described the thickness of the macular GCL in children, but the majority are performed with different OCT techniques [16, 17]. Arnljost et al. reported group of Swedish 6.5 year-old children using the Cirrus HD-OCT device [6]. The mean average GCL thickness was 85.9 μm (sd:5.3) which is thicker than in the present study however due to different devices used the results are not comparable.

Lee et al. presented retrospective data of macular GCL thickness measured with swept source OCT and in their group of healthy Korean children between 3 and 17 years old, the mean average thickness was 71.6 μm range: 49.3–87.2 [7]. The refraction in the group varied from − 12 to + 7.75 and a multivariate analysis showed a correlation with refraction, making the results difficult to compare to the present study. Cheng et al. have used the same OCT technique in a large cohort study with healthy Chinese children [8]. They presented the results as mean average GCL thickness in the macula as well as in the ETDRS charts and compared to the present study those children had a thicker average macular GCL. The refraction in their study had a range of − 11.38 to + 8.38 and they found a correlation between thickness and refraction, where higher myopia was correlated to thinner GCL. This was confirmed in a Spanish study of children with different refraction where the myopic children had significantly thinner GCL thickness than the emmetropic and hyperopic children [17]. In the present study we excluded children with high hyperopia and myopia and hence took away the effect of refraction but still we found a thinner GCL than the large Asian studies.

Previous studies measuring total macular thickness in children has shown a difference according to ethnicity [18]. A more recent study in a large adult Asian cohort showed a significant difference in macular GCL thickness in different ethnic groups where Chinese adults had in average 3.3 μm thicker GCL than adults from Indian heritage [19]. It is possible that different ethnicity could be an explanation to the difference in average thickness in the different studies performed with the same type of OCT device. Our narrow inclusion criteria of +/− 3D may account for this disparity compared to the Asian studies with our participants on average being slightly hyperopic.

In the present study almost, all children were able to perform at least two and often three macula OCT-measurements and the repeatability between the different measurements were particularly good. Similar results were presented by Muñoz-Gallego et al. however using spectral domain OCT [20]. This indicates that OCT measurements of GCL in the macula is a reliable method and well tolerated in children. The GCL is demonstrating a link between morphology and function when it comes to visual field sensitivity [10]. In children where visual field testing can be challenging a repeated examination over time can be of diagnostic and therapeutical value in patients with optic or central nervous system diseases affecting the visual pathway. This can further make intervention possible before subjective detectable function loss is apparent. Possible diseases suitable to may be optic pathway gliomas as showed by Gu et al. [21] or for screening purposes in neurofibromatosis. Neurologic lesions to the central nervous system affecting the visual pathway can hypothetically be visualized in the retina of children as with adults. In the future modern approaches such as deep learning algorithms can possibly be used to predict loss of central visual field from macular OCT cubes in diseases affecting the visual pathways [22].

## Conclusions

In this study we present normative data of macular GCL in healthy children with normal refraction, spherical equivalent mean:1.13 (sd:0.66) dioptre and normal visual acuity Logmar, mean: 0,015 (sd:0,05) using swept source OCT. The OCT measurements of macula GCL showed good CV and ICC.

The results show reliable measurements which is easy to perform for most children. Further studies will show how we can use the information obtained from macular GCL in diagnosis and follow up of different diseases. Updated normative data for comparison, suited for the intended population of interest, is paramount. We also propose additional fixation targets adapted for children in OCT-devices to increase interest and fixation time during examination.

## Data Availability

The datasets used and/or analyzed during the current study are available from the corresponding author on reasonable request.

## Abbreviations

CV: Coefficient of variation
GCL: Ganglion cell layer
GCC: Ganglion cell complex
ICC: Intraclass correlation
ILM: Inner limiting membrane
INL: Inner nuclear layer
IPL: Inner plexiform layer
OCT: Optical coherence tomography
RNFL: Retinal nerve fibre layer
RPE: Retinal pigment epithelium
SE: Spherical equivalent
